# Novel Design Approaches in the Fabrication of Polymeric Microarray Patches via Micromoulding

**DOI:** 10.3390/mi11060554

**Published:** 2020-05-30

**Authors:** Inken K. Ramöller, Emma McAlister, Abigail Bogan, Ana S. Cordeiro, Ryan F. Donnelly

**Affiliations:** School of Pharmacy, Queen’s University Belfast, Medical Biology Centre, 97 Lisburn Road, Belfast BT9 7BL, UK; iramoeller01@qub.ac.uk (I.K.R.); emma.mcalister@qub.ac.uk (E.M.); abogan02@qub.ac.uk (A.B.); a.cordeiro@qub.ac.uk (A.S.C.)

**Keywords:** microarray patch, microneedle, transdermal, polymeric, hydrogel-forming, micromoulding, geometry, ibuprofen sodium

## Abstract

The focus on novel systems for transdermal delivery of therapeutic agents has increased considerably over recent years, as this administration route comes with many advantages. Polymeric microarray patches (MAPs) are minimally invasive devices that enable systemic delivery of a wide range of drugs by overcoming the outer skin barrier. Conventionally, MAPs fabricated by micromoulding have a low needle density. In this study, the performance of hydrogel-forming MAPs cast using novel industrially manufactured micromoulds with a high needle density (600 needles/0.75 cm^2^) was compared to that of MAPs obtained using conventional moulds with a lower density (196 needles/0.89 cm^2^). Surrounding holders for micromoulds were designed for time-efficient fabrication of MAPs. The influence of needle densities on mechanical strength, insertion efficiency and in vitro permeation of ibuprofen sodium (IBU) was analysed. Insertion of both MAPs into an artificial skin model and neonatal porcine skin was comparable. No significant difference was observed in permeation studies of IBU (*p* > 0.05), with a delivery of 8.7 ± 1.7 mg for low-density and 9.5 ± 0.1 mg for high-density MAPs within 24 h. This highlights the potential of these novel micromoulds for manufacturing polymeric MAPs with a higher needle density for future applications.

## 1. Introduction

The focus on transdermal drug delivery systems is increasing considerably as they have several advantages compared to other commonly used administration routes. Variations within the gastrointestinal absorption and hepatic first-pass metabolism, common in the oral administration of therapeutic agents, can be avoided [1]. Parenteral administration, on the other hand, often requires skilled personnel and injections can lead to complications such as pain, haematoma, bleeding and nerve injuries if applied incorrectly [2]. This is why approximately 10% of the world population are affected by needle phobia [3]. Non or minimally invasive transdermal drug delivery systems can overcome these issues. However, due to the nature of skin, only a few therapeutic agents are currently available as transdermal delivery systems in the form of transdermal patches. To overcome the outer barrier of the skin, the stratum corneum, and reach the systemic blood circulation, drugs need to have specific physicochemical properties, such as low molecular weight and lipophilicity [4]. Therefore, recent research has focused on novel delivery devices that can disrupt this barrier.

Microarray patches (MAPs) are minimally invasive transdermal devices composed of multiple micron-scale needles arranged in an array. The needles can vary in terms of geometry and number but they are most commonly below 1 mm in length. This enables them to bypass the outer skin barrier without reaching blood vessels and nerves located in deeper skin layers. Thus, pain or bleeding do not occur. Increasing evidence is displayed in the literature of the great promise of MAPs to enhance the transdermal delivery of both low- and high-molecular-weight therapeutic agents [5,6,7,8,9,10,11,12,13,14]. Research has focused on different types of MAPs made from a wide range of materials. MAPs can be solid, hollow, coated, dissolving or hydrogel-forming. Depending on the type and the intended purpose, materials such as silicon, metals, ceramics, glass, carbohydrates or polymers can be used [15]. While most of these materials are not biocompatible and come with safety issues, polymers have the advantages of being versatile, cost effective, readily available, often biocompatible and biodegradable. Furthermore, depending on the type of polymer used, they can dissolve or form a hydrogel upon skin insertion and control the drug release [16]. They are also self-disabling, meaning that they cannot be reused, reducing the risk of needle stick injuries and spreading infections [15]. Hydrogel-forming MAPs contain no drug but act initially as tools that pierce the stratum corneum. Upon skin insertion, they form a hydrogel by taking up interstitial fluid and, thus, create aqueous conduits between the dermal microcirculation and an attached drug reservoir [17].

Polymeric MAPs can be fabricated using a wide range of techniques. Examples are moulding techniques such as micro, injection and investment moulding or lithography methods [16]. The most commonly used method for polymeric MAPs is micromoulding. This is because a large number of MAPs can be easily produced from one single micromould [16]. Fabrication steps include production of MAP micromoulds (mostly made from silicone by laser drilling or injection moulding), casting of polymer solutions, elimination of air bubbles by application of pressure or centrifugation, solidification and removal from micromoulds. This procedure can be employed both in laboratory surroundings and up-scaled for industrial purposes.

In this study, industrially produced micromoulds of a novel design were used for the first time to cast hydrogel-forming MAPs with a high needle density (600 needles/0.75 cm^2^, “high-density MAPs”). The impact of the needle density on the mechanical strength and insertion efficiency of these MAPs was investigated and compared to MAPs obtained from conventionally used micromoulds with a lower needle density (196 needles/0.89 cm^2^, “low-density MAPs”). Furthermore, the influence of the needle density on the *in vitro* permeation of a low-molecular-weight model drug, namely ibuprofen sodium (IBU), was studied. To allow for a more reproducible and time-efficient fabrication, micromoulds were fitted to custom-made holders, newly designed in house and obtained from 3D-printed templates. The advantages of using these mould–holder combinations, compared to flat micromoulds only, were investigated.

## 2. Materials and Methods

### 2.1. Materials

Xiameter^®^ RTV-4250-S silicone base (white) and curing agent (green) were purchased from Notcutt (Surrey, UK), transparent LSR9-9508-30 silicone elastomer mix from Polymer Systems Technology (High Wycombe, UK) and poly(lactic acid) from Ultimaker (Geldermalsen, The Netherlands). Poly(ethylene glycol) 10,000 Da, anhydrous sodium carbonate, anhydrous monobasic potassium phosphate and ibuprofen sodium were obtained from Sigma-Aldrich (St. Louis, MO, USA). Gantrez^®^ S-97, a copolymer of methyl vinyl ether and maleic acid, 1,500,000 Da was kindly donated by Ashland (Kidderminster, UK). Cryogel^®^ SG/3 (gelatine) was purchased from PB Gelatins GmbH (Nienburg/Weser, Germany), Pearlitol 50 C (mannitol) from Roquette (Lestrem, France), phosphoric acid 85% from Amresco Inc. (Solon, OH, USA) and acetonitrile ≥ 99.9% from Honeywell Research Chemicals (Bucharest, Romania). All other chemicals used were of analytical reagent grade.

### 2.2. Design of Micromoulds and Fabrication of Holders

**Figure 1:**
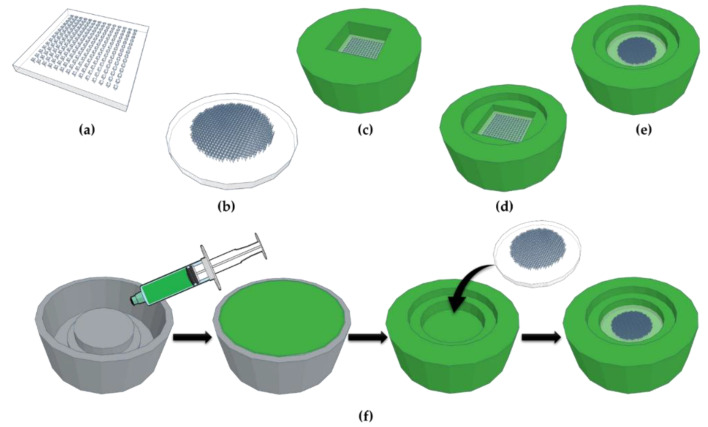
Schematic representation of the different types of micromoulds and mould–holder combinations used: (**a**) industrially produced conventional flat micromould for low-density microarray patches (MAPs), (**b**) industrially produced novel flat micromould for high-density MAPs, (**c**) conventional micromould in commonly used holder (first generation), (**d**) conventional micromould in newly designed holder with a levelled design (second generation), and (**e**) novel micromould in newly designed holder with a levelled design (second generation). (**f**) Holders were cast out of silicone using a 3D-printed template and flat micromoulds were attached to holders with silicone to obtain mould–holder combinations.

Different types of silicone MAP moulds were tested for casting hydrogel-forming MAPs. Flat micromoulds of two different geometries, industrially produced by injection moulding, were used (Table 1). Previously used micromoulds produced square arrays with a low needle density (for example, 11 × 11, 14 × 14 or 19 × 19 needles per array) [9,11,12]. In this study, the properties of conventional micromoulds with an array of 196 (14 × 14) pyramidal shaped needles per 0.89 cm^2^ (height 600 µm, base 350 × 350 µm, interspace 350 µm; Figure 1a) for producing low-density MAPs were compared to novel micromoulds with a higher needle density in a circular area (600 pyramidal needles per 0.75 cm^2^, height 750 µm, base 300 × 300 µm, interspace 50 µm; Figure 1b) for producing high-density MAPs.

To facilitate different casting methods, flat micromoulds were attached to holders of two different designs (Figure 1c–e). Master templates for holders were designed using a computer-aided design software and printed out of poly(lactic acid) with an Ultimaker 3 3D printer (Ultimaker, Geldermalsen, The Netherlands) using Cura^®^ software. Xiameter^®^ silicone base was mixed thoroughly with its green curing agent (10:1 *w*/*w*), cast into the 3D-printed templates and cured at room temperature for approximately 18 h. After removal from templates, flat micromoulds were attached to the holders using a small amount of a transparent LSR9-9508-30 silicone elastomer mix (Part A: Part B 1:1 *w*/*w*), followed by curing at 80 °C for 20 min (Figure 1f). Flat micromoulds alone and mould–holder combinations of two different designs were compared in terms of their feasibility to produce uniform hydrogel-forming MAPs in a reproducible fashion.

### 2.3. Fabrication of Hydrogel-Forming Microarray Patches

An aqueous blend containing 20% *w*/*w* Gantrez^®^ S-97, 7.5% *w*/*w* poly(ethylene glycol) 10,000 Da and 3% *w*/*w* sodium carbonate was prepared for the fabrication of hydrogel-forming MAPs [18]. Approximately 0.8 g of this blend was cast into each mould, followed by application of a pressure of 5 bar in a Protima AT10 pressure tank (Richmond Scientific, Lancashire, UK) for 15 min for flat micromoulds or centrifugation at 5000 rpm for 15 min for mould–holder combinations. Cast MAPs were then dried at room temperature for 48 h before polymers were cross-linked by heating at 80 °C for 24 h. After demoulding of cross-linked MAPs, sidewalls formed during the drying process were either removed using a heated scalpel (first-generation mould–holder combination) or MAPs were carefully taken out of the outer polymer ring manually (second-generation mould–holder combination). MAPs were visually inspected using either a Leica EZ4W stereo microscope with an integrated camera (Leica Microsystems, Milton Keynes, UK) or a Tabletop TM 3030 scanning electron microscope (Hitachi, Tokyo, Japan).

### 2.4. Determination of Mechanical Characteristics and Microarray Patch Insertion

For evaluation of mechanical strength, MAPs were attached to the cylindrical probe (cross-sectional area 1.5 cm^2^) of a TA.XT2 Texture Analyser (Stable Micro Systems, Haslemere, UK) in compression mode. This was moved vertically downward at a speed of 0.5 mm/s, compressing MAPs against a flat aluminium surface with a force of 32 N/array for 30 s. Before and after compression, the length of individual MAP needles was measured using the light microscope and percentage reduction in needle heights was calculated.

The insertion of MAPs into an artificial skin model consisting of eight Parafilm M^®^ layers (Bemis Company Inc., Soignies, Belgium) was evaluated following a method developed and validated by Larrañeta et al. [19]. Briefly, a similar setup as for mechanical characterisation was used (speed 0.5 mm/s), but MAPs were compressed against the artificial skin model instead of an aluminium block. Different forces (32 N, 20 N, 10 N) were applied for 30 s before MAPs were moved upward again and Parafilm M^®^ layers were separated. Holes created in each layer were counted using the microscope and the influence of different forces on insertion depths of low- and high-density MAPs was compared.

Additionally, MAPs were inserted into full-thickness neonatal porcine skin, obtained from stillborn piglets. Neonatal porcine skin is a widely accepted skin model due to its similarities to human skin regarding its general structure, thickness, hair follicle content, pigmentation, collagen and lipid composition [20]. Piglets were stored immediately after birth at −20 °C. Two days before skinning, piglets were defrosted and the obtained full-thickness skin was cut into pieces and again stored at −20 °C until further use. After equilibration in phosphate-buffered saline (PBS, pH 7.4, 30 min), skin samples were shaved, placed on a flat surface and MAPs were inserted manually (force held for 30 s) into the skin. Optical coherence tomography (OCT, EX1301 OCT microscope, Michelson Diagnostics, Kent, UK) was used for visualisation of MAP insertion.

### 2.5. Preparation of Lyophilised Ibuprofen Sodium Wafer-Like Reservoirs

Lyophilised IBU wafer-like reservoirs were prepared as previously described [18]. In brief, gelatine (10% *w*/*w*), mannitol (3% *w*/*w*) and IBU (40% *w*/*w*) were mixed with deionised water in a DAC 150 FVZ-K SpeedMixer™ (Synergy Devices Ltd., High Wycombe, UK) at 3000 rpm for 60 s and sonicated at 37 °C for 60 min. Approximately 250 mg of the resulting formulation was cast into open-ended cylindrical moulds (diameter 13 mm, depth 3 mm) and placed into a Virtis Advantage^®^ Bench top Freeze Drier System (SP Scientific, Gardiner, NY, USA) to be lyophilised. The freeze-drying cycle followed a previously reported protocol [9]. After lyophilisation, reservoirs were visually inspected for uniformity and stored at 2–8 °C until further use.

### 2.6. Pharmaceutical Analysis of Ibuprofen Sodium

A reversed phase high-performance liquid chromatography (RP-HPLC) method was developed with isocratic elution to analyse IBU in PBS (pH 7.4) following *in vitro* permeation studies. The method was achieved on an Agilent 1200 series system (Agilent Technologies UK Ltd., Stockport, UK) and Chemstation^®^ computer software B.02.01 was used for chromatogram analysis. The temperature of the Agilent Zobrax Eclipse XDB-C_18_ column (150 mm length, 4.6 mm internal diameter, 5 µm particle size; Agilent Technologies UK Ltd., Stockport, UK) was maintained at 20 °C. The mobile phase consisted of a 0.02 M solution of monobasic potassium phosphate (pH 2.8) and acetonitrile in the ratio of 30:70 *v*/*v*. The flow rate was kept at 1 mL/min and the injection volume was 50 µL. Each sample was run for 10 min and detected with an ultraviolet detector set at 220 nm. Standard samples of IBU (0.08–50 µg/mL) were prepared in PBS (pH 7.4).

The RP-HPLC method was validated according to the guidelines of the International Conference on Harmonisation (ICH) [21]. The assessed parameters were specificity, linearity, range, accuracy, precision, limit of detection (LoD) and limit of quantification (LoQ). The LoD and LoQ were determined using the standard deviation (SD) of the response and the slope of the calibration curve. One representative calibration plot was generated from subsequently collated plots and least squares linear regression analysis and correlation analysis were performed.

### 2.7. In Vitro Permeation of Ibuprofen Sodium

The *in vitro* permeation of IBU from lyophilised wafer-like reservoirs through low- and high-density hydrogel-forming MAPs across dermatomed neonatal porcine skin was investigated using a modified Franz cell apparatus setup, as described previously [18]. Skin was obtained as aforementioned but trimmed to a thickness of 350 µm using an Integra^®^ Padgett^®^ Electric Slimline Dermatome Model S (Integra^®^ LifeSciences Corporation, Plainsboro, NJ, USA). Circular sections of shaved skin were secured to the donor compartments of the diffusion cells with the stratum corneum facing upwards, using cyanoacrylate glue (Loctite^®^, Dublin, Ireland). These were then placed upon an aluminium foil covered sheet of dental wax for skin support and MAPs were inserted manually into the skin by applying firm pressure with a 1 mL syringe plunger for 30 s. One lyophilised drug-loaded wafer was placed on top of each inserted MAP. An aliquot (20 µL) of PBS (pH 7.4), added beforehand, promoted adhesion of the drug containing reservoir. A 5.0 g stainless-steel cylinder (diameter 11.0 mm) was placed on top of the lyophilised wafer to prevent expulsion of MAPs from the skin. The donor compartments were positioned upon acceptor compartments containing 12 mL PBS (pH 7.4), thermostated at 37 ± 1 °C and stirred at 600 rpm. Each Franz cell was sealed with Parafilm M^®^ to prevent evaporation. Samples (200 µL) were taken at pre-determined time points using 1 mL syringes attached to 8 cm stainless steel needles and replaced immediately with an equal volume of PBS (pH 7.4). Sample IBU content was determined using the validated RP-HPLC method.

### 2.8. Statistical Analysis

All data were expressed as the mean ± SD. Least squares linear regression analysis, correlation analysis, LoD and LoQ were performed using Microsoft Office 365 ProPlus Excel (Microsoft Corporation, Redmond, WA, USA). Statistical analysis was performed using GraphPad Prism^®^ 7 (GraphPad Software, San Diego, CA, USA).

## 3. Results

### 3.1. Characteristics of Micromoulds, Mould–Holder Combinations and Fabricated Microarray Patches

**Figure 2:**
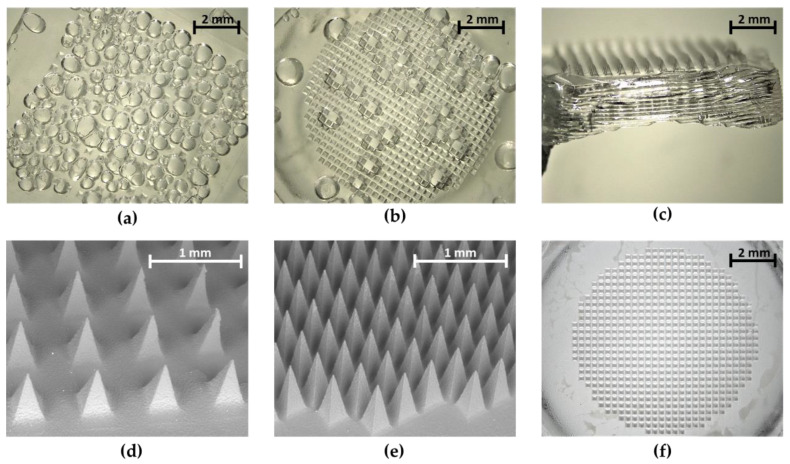
Microscopic images of hydrogel-forming microarray patches (MAPs) cast using different silicone moulds. The use of flat micromoulds resulted in only partly formed MAPs for both (**a**) conventional low-density and (**b**) novel high-density micromoulds. The use of first-generation mould–holder combinations led to the formation of (**c**) thick sidewalls for low-density MAPs (light microscope, 8×). Newly designed mould–holder combinations (second generation) allowed for the production of uniform (**d**) low-density and (**e**) high-density MAPs (scanning electron microscope, 30×) without (**f**) air bubbles and with even backing layers (light microscope, 8×).

The feasibility of different silicone MAP moulds for a reproducible production of uniform hydrogel-forming MAPs was investigated. The micromoulds used were industrially produced. Conventional micromoulds for producing low-density MAPs were compared to those for casting novel, high-density MAPs (3.6-fold higher needle density). To fabricate MAPs and eliminate air trapped within micromould tips, positive pressure is commonly applied to these flat micromoulds. However, in this study, the pressure applied was not sufficient for removing the trapped air, resulting in only partly formed hydrogel-forming MAPs (Figure 2a,b). Flat micromoulds cannot be centrifuged due to the lack of surrounding supports. Therefore, conventional square low-density micromoulds were attached to previously used holders (first generation) to allow for centrifugation. Even though the use of this mould–holder combination resulted in fully formed MAPs, the drying process slowed down considerably due to the small open surface area. Additionally, the formation of thick sidewalls at the back of MAPs could be observed during the drying process (Figure 2c). These sidewalls could not be easily removed and led to uneven MAP backing layers. Therefore, it is reasonable to suggest here that this might have a negative influence on their insertion capabilities as insertion force cannot be evenly applied to these MAPs. To overcome these issues, new holders were designed (second generation) and assembled with the flat micromoulds. This mould–holder combination has a higher surface area that facilitated shorter periods of drying. Further, due to the levelled design, the formation of thick sidewalls could be reduced. After drying, the narrower inner level of the mould–holder combination was fully filled with formulation which led, thus, to an even backing layer. The outer level was designed in a circular shape to reduce the capillary effects that lead to the formation of sidewalls during the evaporation of water as these are increased in corners. Consequently, sidewalls formed in the outer ring were minimal. Another advantage of this second-generation mould–holder combination was that MAPs could easily be taken out of the outer ring with low manual force, avoiding the time-consuming removal of sidewalls with a heated scalpel. Holders for novel circular high-density micromoulds were designed in a similar approach. The use of second-generation mould–holder combinations led to fully formed low- and high-density MAPs with even backing layers (Figure 2d–f). As a result, only MAPs cast using second-generation mould–holder combinations were taken forward for further studies.

### 3.2. Determination of Mechanical Characteristics and Microarray Patch Insertion

Compression of MAPs against a flat aluminium surface with a force of 32 N/array resulted in a reduction in the height of needles for both low- and high-density MAPs. While the height of low-density MAP needles was reduced by 6 ± 4% (*n* = 66, 22 individual needles measured on three different MAPs), high-density MAP needles were only compressed by 2 ± 2% (*n* = 66).

**Figure 3:**
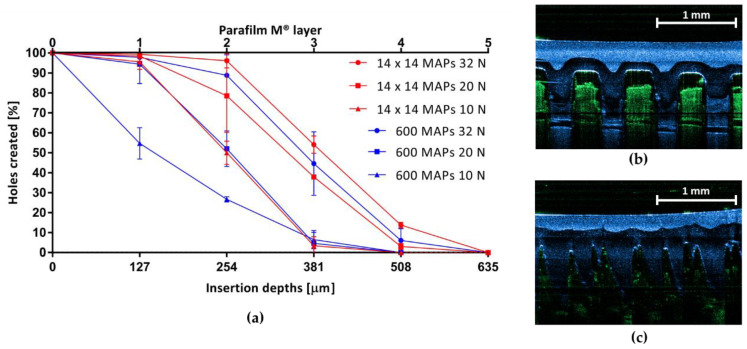
Insertion properties of low- and high-density microarray patches (MAPs) were tested with (**a**) an artificial skin model consisting of eight Parafilm M^®^ layers. The graph shows the percentage of holes (based on the total number of needles per MAP) created in each Parafilm M^®^ layer at different forces (means ± SD, *n* = 3). Insertion of (**b**) low-density and (**c**) high-density MAPs into neonatal porcine skin was assessed. The displayed exemplar images were obtained using optical coherence tomography (artificial colours applied using Image J^®^ (National Institutes of Health, Bethesda, MD, USA)).

Insertion efficiency into an artificial skin model consisting of eight Parafilm M^®^ layers was tested at 32 N, 20 N and 10 N. The percentage number of holes created by the two different MAP types in each Parafilm M^®^ layer at three different forces is displayed in Figure 3a. Even though low-density MAPs showed similar insertion for all three forces into the uppermost Parafilm M^®^ layer (*p* > 0.05), insertion efficiency into deeper Parafilm M^®^ layers decreased significantly (*p* < 0.05) at lower forces. The latter was also the case for high-density MAPs. At 32 N, no significant difference between the insertion of low- and high-density MAPs was observed. A difference only became apparent at lower forces, with better insertion obtained for low-density MAPs. Both MAP types were able to pierce the first four layers of Parafilm M^®^ at 32 N (*n* = 3). With a mean thickness of 127 µm for each layer, this equals an insertion depth of 508 µm, which suggests that low-density MAPs were approximately inserted up to 85% of their total needle length of 600 µm and high-density MAPs up to 68% (total needle length 750 µm).

Analysis of OCT images (Figure 3b,c) showed that both low- and high-density MAPs could be successfully inserted into neonatal porcine skin.

### 3.3. Pharmaceutical Analysis of Ibuprofen Sodium

A RP-HPLC method for the quantification of IBU in PBS (pH 7.4) was developed and validated according to the ICH guidelines. The retention time for IBU was 3.5 min. IBU validation parameters are documented in Table 2.

### 3.4. In Vitro Permeation of Ibuprofen Sodium

**Figure 4:**
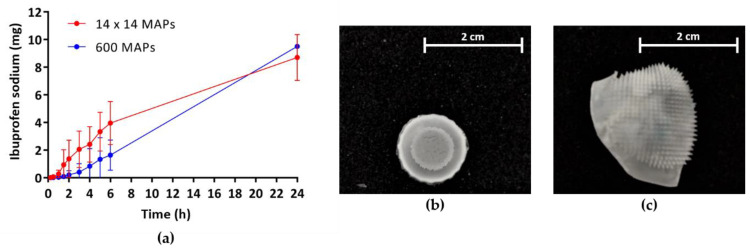
(**a**) *In vitro* permeation profiles of ibuprofen sodium via hydrogel-forming microarray patches (MAPs) (means ± SD, *n* = 3). Microscopic images of high-density MAPs (**b**) before and (**c**) after *in vitro* permeation studies.

The *in vitro* permeation of IBU across neonatal porcine skin commenced within the first 15 min after application of hydrogel-forming MAPs (Figure 4a) and inserted needles remained in the skin for the duration of the experiment. The mean amount of IBU found in the receiver compartments of Franz cells after 24 h was 8.7 ± 1.7 mg (*n* = 3) for low-density MAPs and 9.5 ± 0.1 mg (*n* = 3) for high-density MAPs. However, this difference was not significant (*p* > 0.05). After the *in vitro* permeation experiment, MAPs could be removed intact and had considerably increased in size due to the uptake of PBS (pH 7.4) and the formation of a hydrogel (Figure 4b,c).

## 4. Discussion

The concept of MAPs for transdermal delivery of therapeutic agents was first described in 1976 [22] but only in the late 1990s, advancements in microfabrication technology allowed for researchers to focus on the development and manufacture of these novel devices [23]. Since then, research has shifted from simple “poke and patch” approaches, in which the barrier of the stratum corneum is disrupted by application of solid MAPs and a drug-loaded vehicle is applied after removal of MAPs [23], to more advanced systems like syringe-type hollow or drug-loaded polymeric MAPs. However, to-date, only pen- and syringe-type systems with micron-scale needles have been commercially approved [24]. MAPs made from polymeric materials have a promising future because of the excellent biocompatibility, biodegradability, low toxicity, strength and low cost of these materials [25,26]. Polymers are mainly used for producing MAPs of the dissolving or hydrogel-forming type. With these systems, drug release can be tailored to the desired pharmacokinetic profile and upon removal, due to their self-disabling nature, MAPs cannot be reinserted [15].

The most common technique for the manufacture of polymeric dissolving or hydrogel-forming MAPs is micromoulding. This approach allows for casting multiple arrays using one micromould at both laboratory and industrial scale [16]. In previous studies, square silicone moulds with a low needle density were used. These moulds produced arrays with 11 × 11, 14 × 14 or 19 × 19 micron-scale needles [9,11,12]. In this study, hydrogel-forming MAPs were fabricated using these conventional micromoulds (14 × 14 pyramidal needles, area 0.89 cm^2^, height 600 µm, base 350 × 350 µm, interspace 350 µm) but also novel micromoulds with a higher needle density. The resulting MAPs consisted of 600 pyramidal needles in a circular setup (area 0.75 cm^2^, height 750 µm, base 300 × 300 µm, interspace 50 µm). The aim was to investigate the impact of needle density on the mechanical strength and insertion efficiency of MAPs and on their ability to deliver the low-molecular-weight model drug IBU in an *in vitro* setting.

Conventional and novel micromoulds used were fabricated industrially by injection moulding. In contrast, micromoulds used within our research group in the past were produced from silicone sheets by laser drilling [27]. This approach is highly time consuming as each micromould must be produced individually. Additionally, it can only be used for the fabrication of micromoulds for MAPs with conical needles. Due to the process, micromoulds display a rough surface and application of pressure after casting of polymer solutions is insufficient for removal of air bubbles. Therefore, laser-drilled micromoulds should be attached to holders with surrounding supports to facilitate centrifugation. In comparison, the advantage of micromoulds produced by injection moulding is that this process can be easily up-scaled, allowing for manufacture of multiple micromoulds within a short period of time. Additionally, the shape of individual needles can be customised by adapting the master template and the even surface area allows for use of pressure as an alternative to centrifugation for air bubble removal.

During the casting of hydrogel-forming MAPs it was observed that application of pressure was inadequate for removing the air trapped within micromould tips and, thus, the cast aqueous blend, resulting in only partly formed MAPs. Thus, flat micromoulds were attached to holders to allow for centrifugation. Obtained MAPs were fully formed, but the low open surface area of this first-generation mould–holder combination increased the drying period. Furthermore, MAPs formed thick side walls while drying. Newly designed holders with a two-level setup overcame both issues. Due to the higher surface area, drying times were comparable to flat micromoulds only and after removal from the mould–holder combination, MAPs could be easily taken out of the outer polymer ring and were immediately ready for further usage.

Both conventional low and novel high-density MAPs displayed high mechanical strength, as this property mainly depends on the type of formulation used and the shape of individual needles [15], which was similar for both MAPs. Due to the higher needle density in novel MAPs, a lower insertion efficiency was expected, according to the “bed-of-nails-effect”. This theory was contradicted in obtained results though, as no significant difference in the insertion efficiency into an artificial skin model could be observed between the two MAPs at 32 N, the maximum mean force a human exerts when inserting MAPs [28]. Only at low forces, insertion of high-density MAPs was significantly lower. This has to be taken into consideration if a patient is unable to exert full force. However, a previous study has shown that both young and elderly people were able to apply MAPs with the same efficiency [29] and successful *in vivo* insertion could be controlled by systems such as pressure-indicating sensor films which provide a feedback mechanism for the user [30]. Both MAPs could also be successfully inserted into neonatal porcine skin. This is an important requirement for drug delivery, because MAPs can only take up interstitial fluid after insertion into skin and only then a hydrogel will be formed through which the therapeutic agent can permeate.

Even though the high-density MAPs display more single micron-scale needles, therefore providing more channels for drug permeation upon insertion, no significant difference in the amount of the model drug IBU delivered after 24 h could be observed. This might be because the drug has to partly saturate the hydrogel before it can be released into the surrounding fluid. As the total needle volume of high-density MAPs (13.5 mm³) is 2.8-fold higher than that of low-density MAPs (4.8 mm³), more drug will be retained within the needles before release into the interstitial skin fluid commences. Interestingly, the trend of the observed permeation profile through high-density MAPs followed a linear zero-order release kinetic, an important aspect in controlled drug administration, and a plateau was not reached within 24 h. Therefore, further studies will have to be conducted to gain an understanding of pharmacokinetic permeation profiles over longer periods of time and the feasibility of using these novel high-density hydrogel-forming MAPs for the sustained delivery of different therapeutic agents.

## 5. Conclusions

This is the first time that hydrogel-forming MAPs have been cast using these industrially produced silicone micromoulds of novel geometry. Regardless of their higher needle density of 600 single micron-scale needles on an area of only 0.75 cm^2^, these MAPs could be efficiently inserted into both an artificial skin model and neonatal porcine skin. Furthermore, the in vitro permeation profile of the low-molecular-weight model drug IBU obtained with these high-density MAPs was comparable to that achieved with conventional MAPs of a lower needle density. Newly designed holders for used micromoulds allowed for a faster and more effective way of production, a highly important aspect in upscaling the fabrication of polymeric MAPs to a commercial level. Future studies will show whether these novel high-density micromoulds can be used for producing hydrogel-forming MAPs for the controlled delivery of therapeutic agents and/or for the development of dissolving polymeric MAPs with high drug loading owing to the increased total needle volume. Another approach could be the benefit of swellable hydrogel-forming polymeric MAPs in diagnostics, as MAPs with this novel design are able to take up more interstitial fluid in comparison to conventional MAPs with fewer needles.

## Figures and Tables

**Table 1:** Comparison between the characteristics of conventionally used micromoulds for producing low-density microarray patches (MAPs) and novel micromoulds for casting high-density MAPs.

Properties	Conventional Micromoulds	Novel Micromoulds
Number of needles	196	600
Setup	Square (14 × 14)	Circular
Area	0.89 cm^2^	0.75 cm^2^
Geometry	Pyramidal	Pyramidal
Height	600 µm	750 µm
Base	350 × 350 µm	300 × 300 µm
Interspace	350 µm	50 µm
Total needle volume	4.8 mm³	13.5 mm³

**Table 2:** Validation parameters for the developed reversed phase high-performance liquid chromatography method for the detection and quantification of ibuprofen sodium (IBU) in phosphate-buffered saline (PBS, pH 7.4).

Analytical method	Range (µg/mL)	Slope	y-intercept	r^2^	LoD (µg/mL)	LoQ (µg/mL)
IBU in PBS (pH 7.4)	0.08–50	90.92	3.47	1.0000	0.07	0.22
